# SURGE-ahead postoperative delirium prediction: external validation and open-source library

**DOI:** 10.1007/s41999-025-01180-5

**Published:** 2025-03-10

**Authors:** Thomas Derya Kocar, Philip Wolf, Christoph Leinert, Simone Brefka, Marina L. Fotteler, Adriane Uihlein, Felix Wezel, Martin Wehling, Nuh Rahbari, Hans Kestler, Florian Gebhard, Dhayana Dallmeier, Michael Denkinger

**Affiliations:** 1 Institute for Geriatric Research, AGAPLESION Bethesda Ulm, Ulm University Medical Center, Zollernring 26, 89073 Ulm, Germany; 2 Geriatric Center Ulm, Ulm, Germany; 3 Institute of Medical Systems Biology, Ulm University, Ulm, Germany; 4 Digihealth Institute, Neu-Ulm University of Applied Sciences, Neu-Ulm, Germany; 5 Department of Orthopedic Trauma, Hand, Plastic and Reconstruction Surgery, Ulm University Medical Center, Ulm, Germany; 6 Department of Urology and Pediatric Urology, University Hospital Ulm, Ulm, Germany; 7 Clinical Pharmacology, University of Heidelberg, Mannheim, Germany; 8 Department of Surgery, Ulm University Hospital, Ulm, Germany; 9 Department of Epidemiology, Boston University School of Public Health, Boston, MA USA

**Keywords:** Delirium prediction, Postoperative delirium, Machine learning, Artificial intelligence, Explainable AI

## Abstract

**Purpose:**

In this prospective external validation study, we examined the performance of the Supporting SURgery with GEriatric Co-Management and AI (SURGE-Ahead) postoperative delirium (POD) prediction algorithm. SURGE-Ahead is a collaborative project that aims to develop a clinical decision support system that uses predictive models to support geriatric co-management in surgical wards. Delirium is a common complication in older adults after surgery, leading to poor outcomes and increased healthcare costs. Early and accurate prediction of POD is crucial for timely intervention and prevention strategies.

**Methods:**

The SURGE-Ahead algorithm utilizes a linear support vector machine model with a comprehensive set of 15 clinical and demographic features. In our validation, we analyzed 173 study participants, of which 50 developed POD.

**Results:**

The study found that the SURGE-Ahead POD prediction algorithm yielded state-of-the-art performance, using only preoperative data, with a receiver operating characteristics area under the curve of 0.86. In addition, the SURGE-Ahead algorithm exhibited good calibration as shown by a Brier Score of 0.14. The algorithm is openly available on GitHub, facilitating its implementation and adaptation to different surgical settings.

**Conclusion:**

Our findings contribute to the development of reliable POD prediction tools, ultimately supporting the improvement of patient care in hospitalized older adults.

**Supplementary Information:**

The online version contains supplementary material available at 10.1007/s41999-025-01180-5.

## Key summary points

**Aim:**

To externally validate the performance of the SURGE-Ahead postoperative delirium (POD) prediction algorithm in older adults undergoing surgery.

**Findings:**

The SURGE-Ahead POD algorithm showed excellent predictive ability (ROC AUC 0.86) and good calibration (Brier Score 0.14).

**Implication:**

This validated algorithm can be readily accessed on GitHub, allowing for easy integration into various surgical environments to enhance patient care for hospitalized older adults.

**Supplementary Information:**

The online version contains supplementary material available at 10.1007/s41999-025-01180-5.

## Introduction

Delirium is a severe and common complication in hospitalized older adults aged 65 and older, leading to poor patient outcomes and increased healthcare costs [1, 2]. Early and accurate prediction of postoperative delirium (POD) is essential for timely intervention and prevention strategies, such as the Hospital Elder Life Program (HELP) [3] and the ‘Patientensicherheit, Wirtschaftlichkeit und Lebensqualität’ (PAWEL) project [4]. Notably, the PAWEL project has recently developed POD prediction models, demonstrating a receiver operating characteristic (ROC) area under the curve (AUC) of 0.82 when considering all available data and 0.79 with exclusive reliance on preoperative data [5]. The ability to predict POD before surgery is especially valuable as it allows clinicians to plan accordingly for high-risk procedures and assign targeted delirium prevention interventions. Similarly, other research groups have developed preoperative POD prediction algorithms, yielding ROC AUCs between 0.79 and 0.80 [6–8]. Recently, a scoping review examined the use of machine learning models for predicting delirium in adult inpatients [9]. The review found that while these models show potential, few models have undergone external validation, and even fewer have been prospectively evaluated in clinical practice. Among externally validated models, two stood out for their high ROC AUC scores: first, a linear model with only 6 features (Age, Neurological Disorders, MMSE, Sleep Disorders, Serum Creatinine, ASA score), with an ROC AUC of 0.92 [10]. However, the same features were not associated with the same predictive performance in the PAWEL dataset [11], indicating that this model may not maintain its performance in out-of-sample data. Second, a Random Forest model that was trained on a large corpus of electronic health record data (8561 patients, 858 features), with an ROC AUC of 0.86, although the authors noted poor calibration as a possible limitation [12, 13].

Within the Supporting SURgery with GEriatric Co-Management and AI (SURGE-Ahead) project [14], our goal is to create a clinical decision support system that provides a dashboard-centric interface, supporting surgical teams in the management of older adults. As part of the SURGE-Ahead project, a linear support vector machine model for POD prediction has been developed using the PAWEL data and a comprehensive set of clinical and demographic features [15]. This model was designed with a focus on performance, ease-of-use, transparency, explainability, and ethical considerations. In an internal validation using a hold-out test set of 176 subjects, the ROC AUC was 0.81.

The transition of machine learning algorithms to real-world clinical settings often encounters the challenge of dataset shift, where the target population may differ from the training data, thus necessitating validation and calibration studies to ensure the model’s accuracy and generalizability [16]. The present study aimed to externally validate the SURGE-Ahead POD prediction model using data from a prospective observational study. Additionally, we investigated the impact of calibration methods on the model’s performance.

## Methods

### Study population

**Fig. 1:**
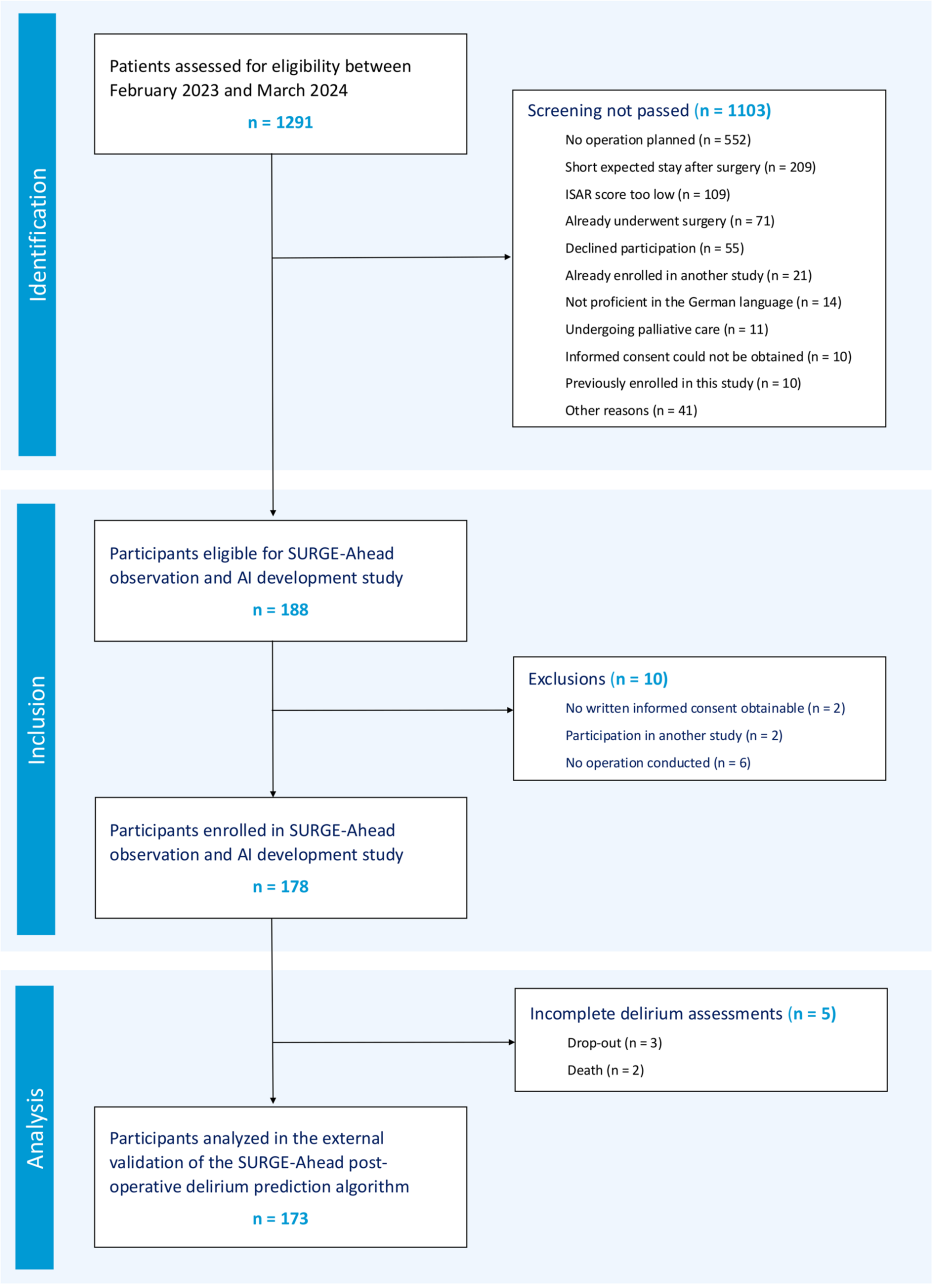
STROBE Flow Chart. After screening 1291 patients, 188 were found eligible and 178 were enrolled in the SURGE-Ahead observation and AI development study. For the present analysis, 5 patients were excluded due to incomplete delirium assessments, resulting in a final cohort of 173 patients

Data for this study were collected during the observation and AI development study of the SURGE-Ahead project [14], which took place between February 2023 and March 2024 at three surgical departments of the Ulm University Medical Center. Out of 1291 screened patients, 178 met the inclusion criteria and were enrolled in the study. All features relevant to the SURGE-Ahead POD prediction algorithm were extracted from the dataset and transformed according to the specifications outlined by Benovic and colleagues [15]. These features were: age (years), estimated glomerular filtration rate (GFR, Cockcroft-Gault, ml/min), ASA class (score), Montreal Cognitive Assessment (MoCA) orientation (subscore), MoCA memory (subscore), number of medications (n), multimorbidity (score), clinical frailty scale (CFS), MoCA verbal fluency (subscore), dementia (Yes/No; determined by preexisting diagnoses and an anesthesiological assessment), recent fall (Yes/No; within the last 3 months), postoperative isolation (Yes/No; due to medical reasons such as multidrug-resistant germs), preoperative benzodiazepines (Yes/No), cardio-pulmonary bypass (Yes/No). The SURGE-Ahead POD prediction algorithm requires the cut-to-suture time, which is only available postoperative. In turn, we obtained procedure-specific cut-to-suture time averages from the three surgical departments at the Ulm University Medical Center, given the OPS code of the procedure. While utilizing procedure-specific averages for cut-to-suture time may overlook interindividual variations, we suggest that this approach still captures the intrinsic procedural risk associated with each surgical intervention. All the input features were either routine electronic health record data, part of a routine standard of procedure, or (in case of the MoCA, fall anamnesis, and CFS) collected by study nurses, not physicians. It is worth noting that the three MoCA subscores necessary for the SURGE-Ahead delirium prediction algorithm can be obtained through the MoCA-5-min assessment proposed by Wong and colleagues (2015), rather than the full-length MoCA test, which could potentially be overly time-consuming for practical implementation [17]. The target variable ‘delirium’ (Yes/No) was determined by repeated 4 ‘A’s tests (4AT) [18] at the postoperative days 1, 3, 5 and 7, each incorporating information from clinical staff and the electronic health record. The 4AT was chosen for its high pragmatism, sensitivity, and specificity, validated by numerous studies including postoperative settings [19, 20]. A test result of 4 or more in any of the postoperative 4AT assessments labeled the subject as positive for delirium. To ensure data quality, each 4AT assessment was supervised by two experienced clinicians (neurologist, geriatrician). Subjects that dropped out of the study before all delirium assessments were conducted were excluded from the analysis, except in cases where delirium had already been confirmed. To identify statistical differences between patients who experienced delirium and those who did not, we employed an exploratory analysis. We utilized the chi-squared test for binary variables, the Mann–Whitney *U* test for discrete variables, and the *t* test for continuous variables when they exhibited a normal distribution as confirmed by the Shapiro–Wilk test. In cases where the continuous variables did not adhere to a normal distribution, we opted for the Mann–Whitney *U* test instead. In total, 173 study participants were included in the present validation and calibration study. For a STROBE flow chart, see Fig. 1 [21]. The study dataset is made publicly available (see the data availability statement).

### External validation

Imputation and normalization of the input data were conducted as outlined by Benovic and colleagues [15]. In brief, missing data were imputed with either the mean, median, or mode of the PAWEL training data, depending on the respective variable. All non-binary data were normalized, with the parameters (mean and standard deviation) calculated from the PAWEL training data. Note that normalizing mean values results in zeroes, which also applies to imputed values. Consequently, imputed values shift the decision function’s output closer to the decision boundary (zero) of the linear support vector classifier. Last, the decision function was calculated via matrix multiplication of the preprocessed data and the model coefficients. All model and preprocessing parameters are presented in Supplementary Table 1. As a performance metric, the ROC AUC was calculated, alongside its 95% confidence interval (CI), determined by bootstrapping with 1000 iterations [22].

### Calibration

The SURGE-Ahead POD prediction algorithm provides a probability estimate obtained by applying a sigmoid transformation (Platt scaling) to the decision function, calibrated on the PAWEL training data [23]. However, since it is unclear if the target demographic of the SURGE-Ahead application (SAA) is comparable to the PAWEL training data, we decided to also recalibrate the model to our study population. We repeated the Platt method (fitting a sigmoid curve) but also applied the Venn-ABERS method, with the help of the open-source ‘venn-abers’ library for Python, provided by Ivan Petej [24]. In essence, the Venn-ABERS method fits an isotonic regression on each class (‘no delirium’, ‘delirium’) and returns the probability estimates (p0, p1) for each class, which can be interpreted as a form of probabilistic prediction interval. In cases where a single probability output is required, this output can be calculated by the formula p = p1/(1 – p0 + p1) [25]. To evaluate the calibration methods, we employed leave-one-out cross-validation [26] and quantitatively assessed the performance of the (re)calibrated models using the Brier Score, which measures the accuracy of probabilistic predictions, and Log Loss, which gages how well the model's predicted probabilities align with the true labels. Additionally, we constructed 95% confidence intervals for these metrics via bootstrapping with 1000 iterations [22]. For visual inspection, a calibration curve with the probability quartiles was plotted, utilizing the 'scikit-learn 1.4.2' library for Python [27].

## Results

### Study population

Out of the 173 patients analyzed in this study, 50 (29%) developed POD, reflecting its incidence in older adults [28]. All features that the model deemed deliriogenic were more severe or more common in patients who developed POD albeit differences in postoperative isolation, preoperative benzodiazepines, and recent falls were not statistically significant. The overall data quality was high, with few missing data: creatinine values were not available preoperatively for all patients, resulting in missing GFR estimations (*n = *19, 11.0%), and the cut-to-suture time could not be estimated for every procedure (*n = *21, 12.1%). In addition, many patients refused the MoCA memory assessment (*n = *38, 22%), or the orientation assessment (*n = *19, 11%). For more details on the study population, see Table 1.

**Table 1:** Study population characteristics

	With Delirium (*n = *50, 28.9%)		Without Delirium (*n = *123, 71.1%)		Group Difference	Total (*n = *173)
Variable	Mean ± SD	*N* (%)	Mean ± SD	*N* (%)	*p*-value	Missing (%)
Demographics						
Age (years)	82.5 ± 6.1		79.8 ± 6.2		< 0.001	0 (0.0%)
Sex (male)		20 (40.0%)		53 (43.1%)	0.839	0 (0.0%)
Sex (female)		30 (60.0%)		70 (56.9%)		
Surgery						
Trauma surgery		39 (78.0%)		90 (73.2%)	0.639	0 (0.0%)
General & visceral surgery		4 (8.0%)		19 (15.4%)	0.289	0 (0.0%)
Urology		7 (14.0%)		14 (11.4%)	0.825	0 (0.0%)
Emergency surgery		32 (64.0%)		59 (48.0%)	0.205	0 (0.0%)
Model features						
ASA class	3.2 ± 0.4		2.8 ± 0.5		< 0.001	1 (0.6%)
I		0 (0.0%)		2 (1.6%)		
II		0 (0.0%)		23 (18.7%)		
III		41 (84.0%)		90 (73.2%)		
IV		8 (16.0%)		8 (6.5%)		
Cardio-pulmonary bypass (Yes/No)		0 (0.0%)		0 (0.0%)		0 (0.0%)
Clinical Frailty Scale (score)	5.4 ± 1.7		3.8 ± 1.7		< 0.001	0 (0.0%)
Estimated Cut-to-suture Time (minutes)	126.0 ± 87.5		99.5 ± 61.5		< 0.001	21 (12.1%)
Dementia (Yes/No)		16 (32.0%)		4 (3.3%)	< 0.001	0 (0.0%)
eGFR (Cockcroft-Gault, ml/min)	52.7 ± 22.1		60.1 ± 23.9		< 0.001	19 (11.0%)
Montreal cognitive assessment					< 0.001	
Memory (subscore)	2.8 ± 1.3		3.2 ± 1.4		< 0.001	38 (22.0%)
Orientation (subscore)	5.2 ± 1.4		5.7 ± 0.9		< 0.001	19 (11.0%)
Verbal Fluency (subscore; binary)		18 (36.0%)		70 (56.9%)	0.020	0 (0.0%)
Multimorbidity (score)	1.7 ± 1.2		1.1 ± 1.0		< 0.001	0 (0.0%)
Number of Medications (*n*)	9.6 ± 4.6		8.5 ± 3.6		< 0.001	0 (0.0%)
Postoperative Isolation (Yes/No)		2 (4.0%)		1 (0.8%)	0.416	0 (0.0%)
Preoperative Benzodiazepines (Yes/No)		8 (16.0%)		12 (9.8%)	0.367	0 (0.0%)
Recent Fall (Yes/No)		36 (72.0%)		70 (56.9%)	0.094	0 (0.0%)

Mean and standard deviation (SD) are reported for continuous and discrete variables, the number of observations (N) for binary variables

*ASA* American Society of Anesthesiologist, *eGFR* estimated glomerular filtration rate

### External validation

**Fig. 2:**
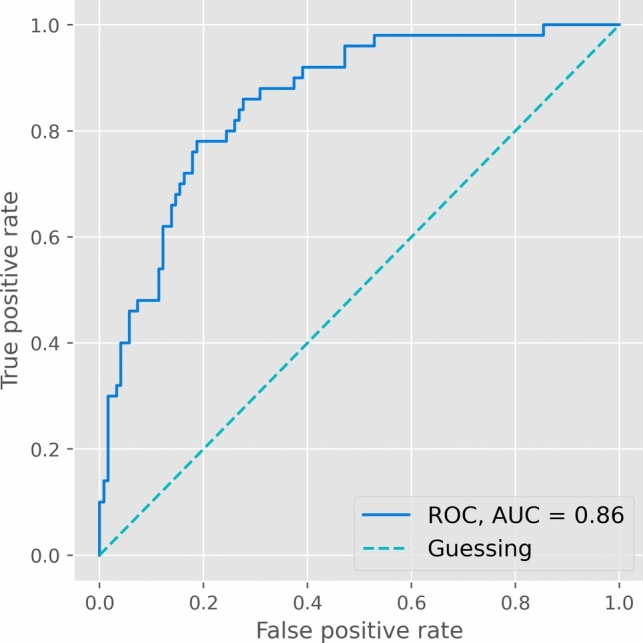
External Validation Receiver Operating Characteristics (ROC). The SURGE-Ahead algorithm achieved an area under the curve (AUC) of 0.86 when externally validated using solely preoperative data

In the external validation of the SURGE-Ahead POD prediction model, the ROC yielded an AUC of 0.86 (95% CI 0.78–0.91; see Fig. 2), indicating state-of-the-art performance.

### Calibration

**Fig. 3:**
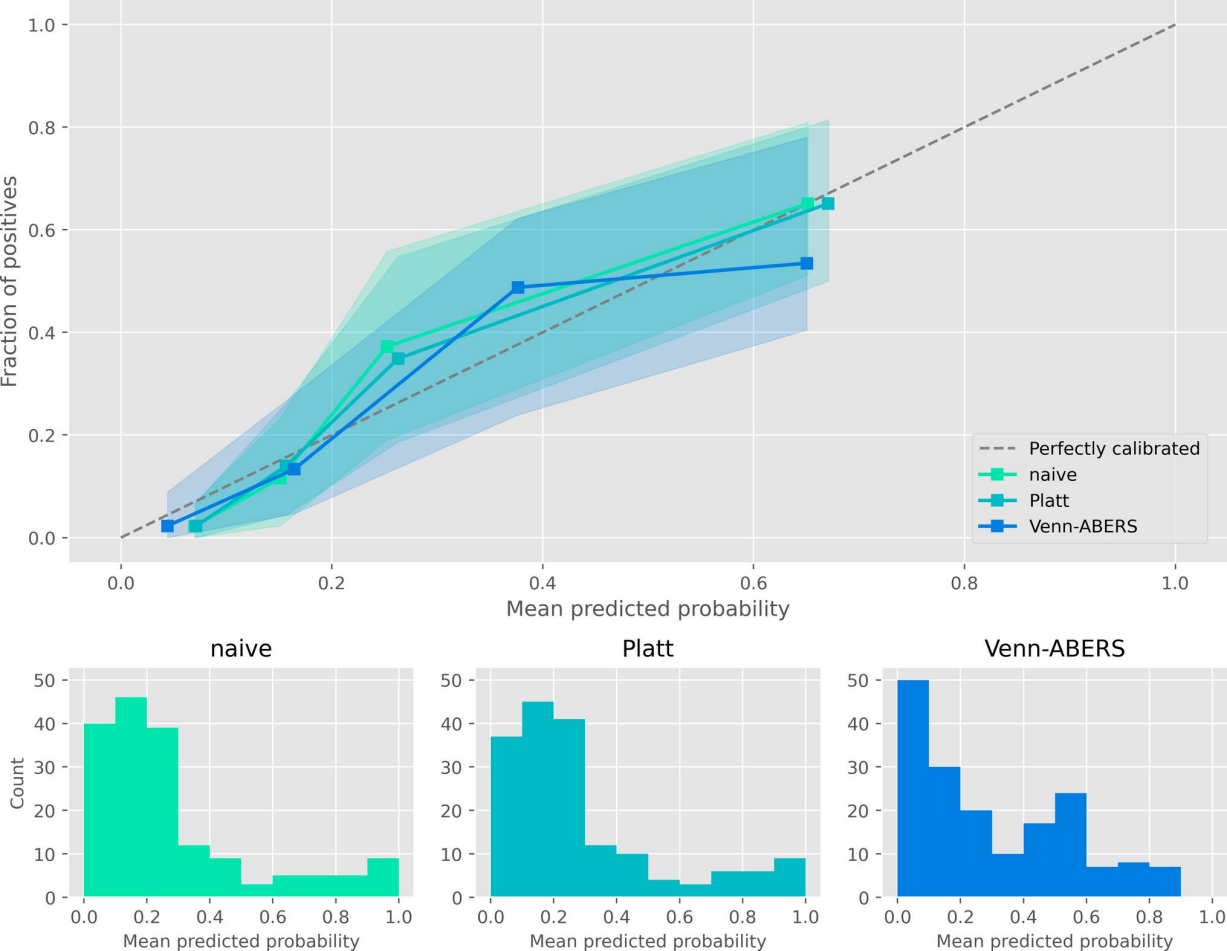
Calibration curves. Top: Calibration plots showing the relationship between predicted probabilities and observed frequencies, along with their respective 95% confidence intervals (shaded areas). Bottom: Histograms illustrating the distribution of probability estimates for each calibration method. Naive: Platt Scaling using the PAWEL dataset. Platt: Recalibration to the SURGE-Ahead dataset using Platt Scaling. Venn-ABERS: Recalibration to the SURGE-Ahead dataset using the Venn-ABERS method

All three calibration methods yielded similar, well-calibrated results. The naive model using Platt calibration on the PAWEL dataset gave a Brier Score of 0.14 (95% CI 0.11–0.18), and a Log Loss of 0.44 (95% CI 0.36–0.53). Recalibrating with Platt Scaling on the SURGE-Ahead demographic resulted in a Brier Score of 0.14 (95% CI 0.11–0.18), and a Log Loss of 0.45 (95% CI 0.37–0.55). The Venn-ABERS method resulted in a Brier Score of 0.15 (95% CI 0.12–0.18), and a Log Loss of 0.45 (95% CI 0.38–0.54). The calibration curve showed good calibration across all quartiles (see Fig. 3).

## Discussion

This prospective observational study externally validated the SURGE-Ahead POD prediction algorithm, achieving a state-of-the-art ROC AUC of 0.86 solely with preoperative data obtained by trained non-physician personnel, which offers a pragmatic implementation option for future use. Moreover, the model demonstrated good calibration to the target population, evidenced by a low Brier Score of 0.14.

Notably, the ROC AUC score obtained in this external validation (0.86) surpassed that achieved during internal validation (0.81) [15]. This discrepancy could suggest either a distribution shift between datasets or potential data quality limitations in the internal validation set. The supplementary results from Benovic and colleagues indicated performance issues in two of the five study centers, while the other centers had an AUC of 0.80–0.86 in the ROC, similar to the present external validation [15]. In either case, the 95% confidence interval of either validation crosses the mean of the other, indicating comparable results.

The SURGE-Ahead POD prediction algorithm, being a linear model, may have resulted in slightly inferior performance compared to non-linear models [13]. However, the choice of a linear model comes with several advantages: (1) Low variance models are generally more robust, which we consider important in healthcare, (2) Linear models are transparent and intrinsically explainable [29], and (3) The implementation of a linear model is straightforward and does not require specific libraries. The decision function can be calculated by a mere dot product, and the feature contributions by element-wise multiplication. Furthermore, the ability to recalibrate the classifier to the target population using the Venn-ABERS method compensates for its marginally lower performance. While it cannot be assured that the SURGE-Ahead POD prediction algorithm will accurately classify each individual patient, conformal prediction provides accurate probability estimation if a sufficiently large calibration set exists. We highly value this assurance offered by the conformal prediction framework because it equips healthcare providers with reliable information, crucial for both practical and ethical decision-making.

Some of the features in the SURGE-Ahead POD prediction algorithm are modifiable risks, such as preoperative benzodiazepines. Pharmacotherapy in older adults poses a health risk in general, but particularly regarding delirium [30]. Our prediction model encompasses only a small dimension of this multifaceted issue and does not consider other deliriogenic drugs. The utilization of tools like the FORTA list, as seen in the broader SURGE-Ahead project, could provide additional value in addressing this limitation [31]. In addition to pharmacotherapy, other modifiable risk factors contributing to delirium include disorientation, pain, immobility, sensory impairment, infections, urinary retention, sleep disturbances, infarctions, and other pathologies [32]. It is essential to acknowledge that there is presently no pharmacotherapy that directly targets the treatment of delirium itself. Nevertheless, once underlying causes are adequately addressed, antipsychotics may be judiciously prescribed based on symptomatology, with due diligence to prevent overmedication [33].

The SURGE-Ahead POD prediction algorithm is published on the project's GitHub page and comes with a library written in Python (https://github.com/IfGF-UUlm/SA_Delirium/). While it is not required to use our library to implement the SURGE-Ahead POD prediction algorithm, using the library comes with several advantages. First, if the user provides the algorithm with a calibration set according to the model specifications, both Platt Scaling and Venn-ABERS calibration can be applied for individual predictions. This even allows for the implementation of a self-learning system, where every new data point can be added to the calibration set and consequently improves calibration. Second, we implemented additional methods of imputation, where new data are imputed according to the calibration set, e. g., using the *k*-nearest neighbor (KNN) method [34]. Third, the library handles several data types, which may simplify implementation in the local environment.

### Limitations

At the time of publishing this article, the SURGE-Ahead POD prediction algorithm has not been approved by any regulatory body for medical devices. The SURGE-Ahead study group is currently working on receiving regulatory approval within the European Union; however, this may take several years to be accomplished. In addition, there are several further limitations to this study: although the data quality of the observation and AI development study was very high, this came at the cost of relatively few samples. While this was less of an issue regarding the external validation, having only 173 (leave-one-out) samples for calibration is suboptimal, especially regarding high-risk patients, of which only a few were in the data. However, it should be noted that the validity guarantees of conformal prediction still hold true, although the probabilistic prediction interval may become wider and harder to interpret. This limitation can also be addressed “on the job”, where every new sample can be appended to the calibration set and in turn improve the calibration. Second, there was a potential overlap between CFS and dementia due to cognitive impairments influencing the CFS assessment. To investigate this, we calculated the mutual information between CFS and dementia, finding it to be low (< 0.08 bits), thus indicating minimal redundancy between these variables in our study [35]. Third, it is unclear whether the SURGE-Ahead POD prediction algorithm improves clinical outcomes. To address this issue, within the SURGE-Ahead project, an intervention study is scheduled for 2025–2026. We believe that timely interventions can reduce the delirium rate and improve other secondary outcomes relevant to the patients’ wellbeing. Last, the observation and AI development study being a single-center study could mean that the external validation may not generalize to other centers. However, we contend that the robustness of linear models will prevent catastrophic failure, and recalibration to the new demographic is always possible.

## Conclusion

We successfully externally validated the SURGE-Ahead POD prediction algorithm in a prospective observational study (ROC AUC 0.86). In addition, we demonstrated accurate out-of-the-box calibration (Brier Score 0.14). The model is made available as an open-source library on the project’s GitHub page, allowing for implementation and recalibration to the local demographic with just a few lines of code: https://github.com/IfGF-UUlm/SA_Delirium/.

## Supplementary Information

Below is the link to the electronic supplementary material.

Supplementary file1 (DOCX 31 KB)

## Data Availability

Data used in this specific analysis can also be accessed on the project’s GitHub page: https://github.com/IfGF-UUlm/SA_Delirium/.
