# Supplementary material for: SURGE-ahead postoperative delirium prediction: external validation and open-source library
Source: Eur Geriatr Med. 2025 Mar 10;16(3):851–9. doi: 10.1007/s41999-025-01180-5 (PMC12174281; doi:10.1007/s41999-025-01180-5)
Supplement: Supplementary file 1 — Supplementary file1 (DOCX 31 KB) [file 41999_2025_1180_MOESM1_ESM.docx]

SURGE-Ahead Postoperative Delirium Prediction: External Validation and Open-Source Library

Supplementary Information

Thomas D Kocar^1,2^, Philip Wolf^3^, Christoph Leinert^1,2^, Simone Brefka^1,2^, Marina L Fotteler^4^, Adriane Uihlein^5^, Felix Wezel^6^, Nuh Rahbari^7^, Hans Kestler^3^, Florian Gebhardt^5^, Dhayana Dallmeier^2,8^, Michael Denkinger^1,2^

^1^ Institute for Geriatric Research at AGAPLESION Bethesda Ulm, Ulm University Medical Center, Ulm, Germany

^2^ Geriatric Center Ulm, Ulm, Germany

^3^ Institute of Medical Systems Biology, Ulm University, Ulm, Germany

^4^ DigiHealth Institute, Neu-Ulm University of Applied Sciences, Neu-Ulm, Germany

^5^ Department of Orthopedic Trauma, Hand, Plastic and Reconstruction Surgery, Ulm University Medical Center, Ulm, Germany

^6^ Department of Urology and Pediatric Urology, University Hospital Ulm, Ulm, Germany.

^7^ Department of Surgery, Ulm University hospital, Ulm, Germany.

^8^ Department of Epidemiology, Boston University School of Public Health, Boston, MA, United States

**Supplementary Table 1: SURGE-Ahead POD prediction model parameters**. To use the model, replace missing values with defaults and normalize variables using mean and standard deviation. Then, compute *z* by adding the product of each preprocessed input value and its coefficient, along the intercept. For a probabilistic output, apply Platt scaling $1/{(1+e^{(Az+B)})}$, or recalibrate the model to the target demographic. GFR = glomerular filtration rate, ASA = American Society of Anesthesiologists, MoCA = Montreal Cognitive Assessment.

| **Feature** | **Unit** | **Coefficient** | **Default** | **Mean** | **SD** |
| --- | --- | --- | --- | --- | --- |
| Cut-to-Suture Time | minutes | 0.53 | 146 | 146.05 | 85.29 |
| Age | months | 0.1 | 934 | 934.47 | 58.86 |
| GFR (Cockcroft-Gault) | ml/min | -0.03 | 69 | 69.21 | 22.39 |
| ASA Class | score | 0.39 | 3 | 2.81 | 0.6 |
| MoCA Orientation | subscore | -0.27 | 6 | 5.85 | 0.56 |
| MoCA Memory | subscore | -0.34 | 2 | 2.24 | 1.66 |
| Number of Medications | n | 0.11 | 6 | 6.09 | 3.42 |
| Multimorbidity | score | 0.14 | 1 | 1.27 | 1.35 |
| Clinical Frailty Scale | score | 0.21 | 3 | 3.62 | 1.37 |
| MoCA Verbal Fluency | subscore | -0.26 | 0 | 2.24 | 1.66 |
| Dementia | Yes/No | 2.53 | 0 |  |  |
| Recent Fall | Yes/No | 0.31 | 0 |  |  |
| Postoperative Isolation | Yes/No | 0.15 | 0 |  |  |
| Preoperative Benzodiazepines | Yes/No | 0.42 | 0 |  |  |
| Cardio-Pulmonary Bypass | Yes/No | 0.53 | 0 |  |  |
| Intercept | constant | -0.61 |  |  |  |
| A (Platt parameter) | constant | -0.97 |  |  |  |
| B (Platt parameter) | constant | 1.07 |  |  |  |
